# An Elevated *IL10* mRNA Combined with Lower *TNFA* mRNA Level in Active Rheumatoid Arthritis Peripheral Blood

**DOI:** 10.3390/cimb46030167

**Published:** 2024-03-20

**Authors:** Georgi Vasilev, Viktoria Vasileva, Mariana Ivanova, Spaska Stanilova, Irena Manolova, Lyuba Miteva

**Affiliations:** 1 Laboratory of Hematopathology and Immunology, National Specialized Hospital for Active Treatment of Hematological Diseases, Plovdivsko Pole Str. No. 6, 1756 Sofia, Bulgaria; 2 Medical Faculty, Sofia University St. Kliment Ohridski, 1 Kozyak Str., 1407 Sofia, Bulgaria; 3 Department of Molecular Biology, Immunology and Medical Genetics, Medical Faculty, Trakia University, Armeiska Str. No. 11, 6000 Stara Zagora, Bulgaria; spaska.stanilova@trakia-uni.bg (S.S.); irena.manolova@trakia-uni.bg (I.M.); lyuba.miteva@trakia-uni.bg (L.M.); 4 Clinical Laboratory, Trakia Hospital, Dunav Str. No. 1, 6000 Stara Zagora, Bulgaria; 5 Clinic of Rheumatology, University Hospital “St. Ivan Rilski”, Urvich Str. No. 13, 1612 Sofia, Bulgaria; mariana_ig@abv.bg; 6 Medical Faculty, Medical University-Sofia, Ivan Geshov Blvd. No. 15, 1431 Sofia, Bulgaria

**Keywords:** blood cells, cytokines, disease activity, FOXP3, mRNA, rheumatoid arthritis

## Abstract

We aimed to investigate the expression of pro-inflammatory cytokine genes *TNFA*, *IL6*, *IL12B*, *IL23*, *IL18* and immunoregulatory genes *FOXP3*, *TGFB1*, and *IL10* in the peripheral blood of patients with rheumatoid arthritis (RA) at messenger ribonucleic acid (mRNA) level. The total RNA was isolated from peripheral blood samples. Real-time quantitative PCR was used to perform TaqMan-based assays to quantify mRNAs from 8 target genes. *IL23A* was upregulated (1.7-fold), whereas *IL6* (5-fold), *FOXP3* (4-fold), and *IL12B* (2.56-fold) were downregulated in patients compared to controls. In addition, we found a strong positive correlation between the expression of *FOXP3* and *TNFA* and a moderate correlation between *FOXP3* and *TGFB1.* These data showed the imbalance of the T helper (Th) 1/Th17/ T regulatory (Treg) axis at a systemic level in RA. In cases with active disease, the *IL10* gene expression was approximately 2-fold higher; in contrast, the expression of *FOXP3* was significantly decreased (3.38-fold). The main part of patients with higher disease activity expressed upregulation of *IL10* and downregulation of *TNFA*. Different disease activity cohorts could be separated based on *IL10*, *TNFA* and *IL12B* expression combinations. In conclusion, our results showed that active disease is associated with an elevated *IL10* and lower *TNFA* mRNA level in peripheral blood cells of RA patients.

## 1. Introduction

Rheumatoid arthritis (RA) is a systemic inflammatory disease causing chronic joint inflammation. The prevalence of RA remains relatively constant in many populations, ranging from 0.5% to 1.0% [1]. Although clinical arthritis is necessary for diagnosis, most RA patients experience joint symptoms before clinical arthritis (joint swelling) develops. Studies have also shown that proinflammatory cytokines can increase months before the diagnosis of RA [2]. The pathogenesis of RA is primarily due to repeated activation of both innate and adaptive immune systems. This activation leads to a breakdown of immune tolerance, abnormal autoantigen presentation, and activation of antigen-specific T and B cells [3]. In patients with disease activity flares, the inflammation inevitably progresses to irreversible articular cartilage and bone damage that can lead to lifelong disability and require an adequate treatment strategy [4]. Although persistent joint inflammation is not a life-threatening condition per se, continuous systemic inflammation accompanied by increased levels of tumour necrosis factor-alpha (TNF-α), interleukin (IL)-6 IL-6, IL-1, IL-12, IL-23, and immune system overstimulation may result in secondary amyloidosis and vascular disease as well [5].A reference to the RA patient’s life expectancy and most frequent causes of death confirms that systemic inflammation and vascular events are closely entangled [6]. Therefore, insightful knowledge and an improved understanding of inflammatory pathways are prerequisites for future therapeutic solutions.

As a constant pro-inflammatory background, the propensity to break autotolerance should be sought in dysregulations in cytokine levels and inter-regulation of innate immune response mechanisms [7]. Several genetic, epigenetic, and acquired factors influence a pro-inflammatory context of RA that enables auto-tolerance breaking. Genetic polymorphisms, including single nucleotide polymorphisms (SNPs) in the regulatory part of the cytokine gene, influence cytokine levels [8,9,10]. Aberrant epigenetic changes in RA, including deoxyribonucleic acid (DNA) methylation pattern, histone modification, and micro ribonucleic acid (miRNA) expression, have already been identified as factors in the pathogenesis and progression of the disease, not only in synovial fibroblasts but also in the peripheral blood of patients [11]. Also, certain cytokine pathways and cytokine levels are increased before the onset of the disease and appear to be a predisposing background. In contrast, others may be increased secondary to the autoinflammation that adds hurdles to obtaining a clear conceptual view of the process.

In addition to the increasingly accumulating evidence for the complexity of gene expression regulation, which may result in poor messenger RNA (mRNA)–protein correlations and tightly connected T helper (Th) 17/T regulatory (Treg) imbalanced cytokine production with RA development and progression [12], we focus our current study on a set of pro-inflammatory and immunoregulatory cytokine genes. In line with the other studies, we have previously reported significantly elevated serum levels of pro-inflammatory cytokines, IL-6, IL-17A, IL-23, TNF-a, IL-18, and IL-12p40 as well as the immunoregulatory IL-10 in RA [13]. Elevated levels of IL-10 have been discovered in the serum and synovial fluid of patients with RA. In RA, an exacerbated up-regulation of IL-10 at the transcriptional level was reported previously [4,14]. IL-10 is the most powerful anti-inflammatory cytokine, and it appears that IL-10 plays a dual role. On the one hand, it suppresses pro-inflammatory cytokines. On the other hand, it enhances the humoral autoimmune response [4]. This could explain why a clinical trial administering anti-IL-10 antibodies to treat another autoimmune disorder—systemic lupus erythematosus—has not yet been validated [15]. More recently, a fusion protein of interleukin-4 and interleukin-10 (IL4-10 FP) was synthesised as a potential immunoregulatory drug [16]. IL-4 is a member of the Th2 family of cytokines, together with IL-5 and IL-13. It was suggested that activation of Th2 cells resulting in IL-4 and IL-13 production are involved in the resolution of inflammation in RA [17]. It is clear that the pathogenesis of RA involves a complex network of various cytokines and cells, and except TNF-α and IL-6, other cytokines such as IL-23, IL-17, IL-10, and transforming growth factor-beta 1 (TGF-b1) also play roles at a systemic or local level [18]. Respectively, blood-based mRNA gene expression has the potential to identify patients suitable for a given treatment regimen. Recent studies suggest cytokine gene expression analyses in different cell compositions of peripheral blood mononuclear cells (PBMCs) as a helpful approach to the efficacy of response to disease-modifying drugs in RA patients [19,20,21].

The present cross-sectional study aimed to analyse the expression of pro-inflammatory cytokine genes *TNFA*, *IL6*, *IL12B*, *IL23*, *IL18* and immunoregulatory genes *FOXP3*, *TGFB1*, and *IL10* in PBMCs of patients with rheumatoid arthritis at mRNA levels in relation with disease characteristics, as well as to identify some potential biomarkers for disease activity in RA.

## 2. Materials and Methods

### 2.1. Study Subjects

In this cross-sectional study, we recruited consecutive patients diagnosed with rheumatoid arthritis who attended the Rheumatology Clinic of University Hospital “St. Ivan. Rilski” in Sofia between October 2021 and October 2022. The inclusion criteria for RA patients were persons aged 18 or older who met the American College of Rheumatology (ACR)/European League Against Rheumatism (EULAR) 2010 RA classification criteria [22]. Clinical assessment included collecting demographic data and obtaining information regarding disease activity, autoantibody status, and therapy for RA. Disease activity in RA patients was assessed using the Disease Activity Score 28, based on C reactive protein (DAS28-CRP) [23]. Exclusion criteria were no history of other inflammatory rheumatological or autoimmune disorders, malignancy, body mass index (BMI) ≥ 29.9, and significant unstable/uncontrolled acute or chronic disease or current active infection and previous treatment with biologic disease-modifying anti-rheumatic drugs (bDMARDs) or Janus kinase inhibitor therapy for RA.

Comparisons were made with healthy controls (HC) that were unrelated to each other and selected from university and hospital staff. The exclusion criteria were the same as mentioned above.

The study was approved by the local Ethics Committee of the University Hospital “St. Ivan Rilski” in Sofia, Bulgaria, with decision number 6, dated 29 November 2016, and the Ethics Committee of the Medical Faculty, Trakia University (protocol 16/19.03.2021).

### 2.2. Blood Samples

Gel/clot activator vacutainer tubes collected blood samples from RA patients and controls. The blood samples were allowed to clot for 30 min at room temperature before being centrifuged. For enzyme-linked immunosorbent assay (ELISA) analysis, serum samples were collected and frozen at −70 °C. Peripheral blood was collected in vacutainer tubes containing ethylenediaminetetraacetic acid (EDTA) as an anticoagulant for RNA isolation.

### 2.3. Reverse Transcription PCR

Reverse transcription PCR and qPCR were done as previously described [24]. First, total RNA was isolated from 500 µL peripheral blood samples. RNA extraction was performed using the GeneJET Whole Blood RNA Purification Mini Kit from Thermo Fisher Scientific Inc. (Waltham, MA USA) according to the manufacturer’s instructions. The RNA quantity was measured using the GeneQuant 1300 spectrophotometer from GE Healthcare Life Sciences, Zürich, Switzerland. RNA purity was evaluated based on the A260/280 ratio, which exceeded 1.9. The cDNA was obtained by reverse transcription PCR using the RevertAid First Strand cDNA Synthesis Kit from Thermo Fisher Scientific Inc. The PCR was performed on a GeneAmp PCR System 9700 from Applied Biosystems (Foster City, CA, USA). The reaction utilised random hexamers as primers for cDNA synthesis with RevertAid reverse transcriptase following the manufacturer’s guidelines.

### 2.4. Real-Time Quantitative PCR

The 7500 Real-Time PCR System from Applied Biosystems, Foster City, CA, USA was used to perform TaqMan-based assays for the real-time quantification of mRNAs from 8 target genes: *FOXP3*, *TGFB1*, *IL10*, *IL23A*, *IL12B*, *TNFA*, *IL6*, and *IL18.* The primers-probes used in the study were pre-designed and inventoried assays manufactured by Thermo Fisher Scientific Inc. The ID numbers of the assays used are as follows: *FOXP3*: Hs01085834_m1; *TGFB1*: Hs00998133_m1; *IL10*: Hs00174086_m1; *IL23A:* Hs00372324_m1; *IL12B*: Hs00233688_m1; *TNFA*: Hs00174128_m1; *IL6*: Hs00985639_m1; and *IL18*: Hs01038788_m1. The glyceraldehyde 3-phosphate dehydrogenase (*GAPDH*) Hs02758991_g1 and eukaryotic 18S ribosomal RNA: Hs999901_s1 were reference genes. Maxima Probe qPCR Master Mix (Thermo Fisher Scientific) was used in a final volume of 25 μL per sample. The technical replicates were carried out for any sample (triplicate wells per sample) to ensure accuracy and consistency. The temperature conditions were according to the manufacturer’s instructions. The process began with an initial denaturation step at 95 °C for 10 min, followed by 40 cycles of denaturation at 95 °C for 15 s and annealing/extension at 60 °C for 60 s. We included negative controls (no template control) in each run as a standard practice. The data were collected using the 7500 Software v.2.3 developed by Life Technology in Foster City, CA, USA.

Relative quantification was analysed using the comparative Ct method, also known as the 2^−ΔΔCt^ method, explained by Livak and Schmittgen [25]. After normalisation to the averaged reference genes, the results are presented as a relative quantity (RQ) or fold change (FC) of the target genes compared to the control group (calibrator).

### 2.5. Quantification of Serum Cytokines Concentrations

Serum levels of IL-6, IL-10, IL-12p40, IL-17A, IL-18, IL-23, TNF-α, and TGF-β1 were quantified using commercially available ELISA kits, following the Invitrogen Corporation and eBioscience’s instructions. IL-6, IL-12p40, IL-23, and TNF-α ELISA kits were purchased from Invitrogen Corporation (Camarillo, CA, USA), and TGF-β1, IL-10, IL-17A, and IL-18 kits were purchased from eBioscience (Vienna, Austria). A standard curve constructed using the kit’s standards was used to determine the concentration of cytokines expressed in picograms per millilitre (pg/mL). Serum samples from patients and controls were analysed in duplicate within the same kit’s batch. The minimum detection levels were below 2.0 pg/mL for IL-6 and IL-12p40, 0.05 pg/mL for IL-10, 0.5 pg/mL for IL-17A, 9 pg/mL for IL-18, 4 pg/mL for IL-23, 0.09 pg/mL for TNF-α, and 8.6 pg/mL for TGF-β1. Values below the detection limits were set as zero.

### 2.6. Data Analysis

Statistical analysis and data visualisation procedures were conducted in Jupyter Notebook (Python 3.11.8) and RStudio version 1.4.1717 (R-programming language for statistical computing). The normality of the distribution of different variables was assessed using the Shapiro–Wilk test due to the relatively small sample sizes. Different groups were compared using the Mann–Whitney exact U test in case of non-normally distributed data; otherwise, Student’s *T*-test was applied. Multiple linear regression analyses were performed to adjust for potential confounding factors, such as sex and age. Correlations between different variables were assessed using Pearson and Spearman tests for correlation. Decision Trees for Classification (CART Analysis) were used to study the nonlinear relationship between cytokine variables. CART Analysis was conducted in RStudio using the “rpart” library in RStudio. The “Seaborn” package was used for the data visualisation (Jupyter Notebook, Python). To estimate the power of mRNA levels in peripheral blood as valuable biomarkers, receiver operating characteristic (ROC) analyses were conducted. The statistical significance level was set at the 5% threshold (alpha = 0.05).

## 3. Results

### 3.1. Study Subjects

The RA group consisted of 31 patients with a mean age of 43.1 years (SD ± 14.2), ranging from 18 to 73 years old. The average disease duration was 9.6 ± 8.5 years. The disease onset was below 40 years in 52% of the patients and above 40 years in 48%. 71% of patients with rheumatoid arthritis were anti-cyclic citrullinated peptide antibody (anti-CCP)-positive, and 81% were rheumatoid factor (RF-IgM)-positive. The disease activity state was categorised as follows: low disease activity/remission with DAS28-CRP < 3.2; moderate activity with DAS28-CRP ≥ 3.2 ≤ 5.1; high activity of the disease with DAS28-CRP > 5.1.

Regarding drug therapy, nine (29%) of the patients received only symptomatic medications (long-term low-dose prednisolone 10 mg/day), while 22 (71%) were treated with the conventional synthetic disease-modifying anti-rheumatic drug [csDMARD)-methotrexate (MTX) 15 mg once weekly orally] with folic acid supplementation 5 mg weekly.

For the control group, 21 healthy individuals (3 men and 18 women) were selected. Their ages ranged from 24 to 73 years, with an average age of 39.3 and a standard deviation of 12.2. All participants were of white Caucasian ancestry.

Demographic characteristics and clinical data of RA patients and control subjects are summarised in Table 1.

### 3.2. Gene Expression at mRNA Level in Peripheral Blood Cells

**Figure 1:**
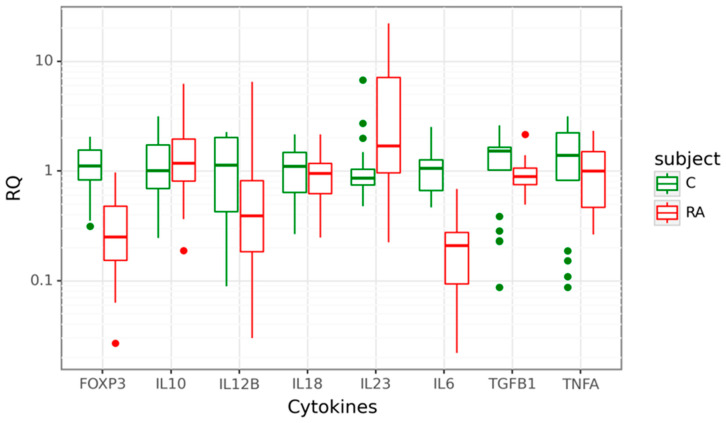
Box plots displaying mRNA expression in relative quantity (RQ) of target genes in 31 rheumatoid arthritis (RA) cases in red and 21 controls (C) in green. The line denotes the median values; boxes, the lower (Q1) and upper (Q3) quartiles; whiskers, minimum and maximum; and single point, outliers. The log scale is used for the Y-axis.

Gene expression at mRNA level was detected for Treg-related genes–*FOXP3, TGFB1*, and *IL10* and genes encoding pro-inflammatory cytokines–*IL23A*, *IL12B*, *TNFA*, *IL17A*, and *IL18* in the cohort of 31 patients diagnosed with RA, compared to 21 age-matched healthy controls. Four of the eight studied target genes showed differential expression between cases and controls. The expression of *IL23A* was 1.7-fold higher in the peripheral blood cells of RA patients compared to controls (*p* = 0.011). In contrast, *IL6, FOXP3*, and *IL12B* mRNA levels were significantly lower in cases. We observed a significant 5-fold decrease of *IL6* mRNA (*p* < 0.001), a 4-fold decrease of *FOXP3* mRNA (*p* < 0.001) and a 2.56-fold decrease of *IL12B* mRNA (*p* = 0.015). There was no significant difference in *IL10*, *IL18*, *TGFB1*, and *TNFA* mRNA expression between the two groups. Data are shown in Figure 1.

The influence of age and sex as positional confounding variables was explored using multivariate linear regression with RQ cytokine levels as a dependent variable and age and sex as regressors or independent variables. We found that only the levels of *TGFB* mRNA and *IL23* mRNA were age-dependent: partial eta squared = 0.18, *p* = 0.002; and partial eta squared = 0.003, *p* = 0.024, respectively. Partial eta squared higher than 0.06 stands for medium to large effect size, and lower than 0.01 indicates small effect size. The effect size of age on *TGFB* mRNA expression is large, and the effect size of age on *IL23* mRNA levels is small. The multivariate linear regression model adjusted for the confounding effect of age and sex showed a non-significant difference in *TGFB* mRNA expression between RA and controls, *p* = 0.2, and up-regulation of *IL23* mRNA in RA patients, *p* = 0.014.

**Figure 2:**
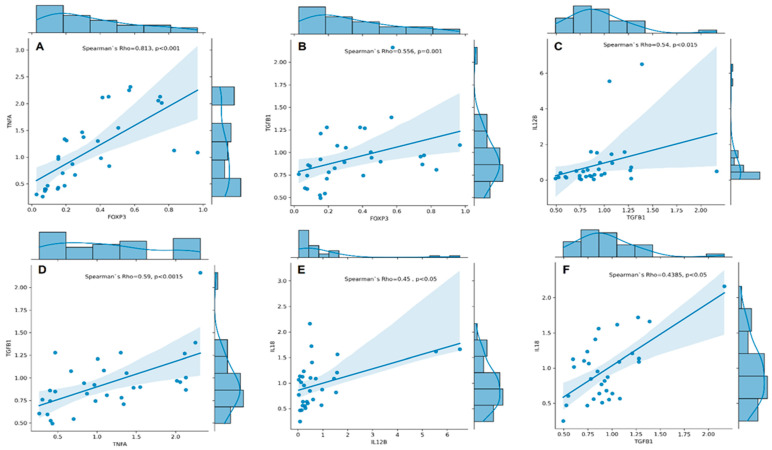
Correlation plots with regression line and marginal densities show correlations between *TNFA* and *FOXP3* (**A**); *TGFB1* and *FOXP3* (**B**); *IL12B* and *TGFB1* (**C**); *TGFB1* and *TNFA* (**D**); *IL18* and *IL12B* (**E**), and *IL18* and *TGFB1* (**F**) mRNA expressions (RQ cytokine levels) in RA patients (n = 31). The histograms on the upper X- and right Y-axes visualise individual cytokine RQ distributions. The Spearman’s rho–coefficient and p-values are presented.

We conducted Spearman’s rank correlation analyses and found a strong positive correlation between the expression of *FOXP3* and *TNFA* (Spearman’s rho = 0.813, *p* < 0.001, Figure 2A). Additionally, we found a moderate correlation between the expression of *TGFB1* and *FOXP3* (r_s_ = 0.556, *p* = 0.001, Figure 2B), *IL12B* (r_s_ = 0.540, *p* = 0.002, Figure 2C), and *TNFA* (r_s_ = 0.590, *p* < 0.001, Figure 2D). Furthermore, our analysis revealed moderate to low correlations between expression of *IL18* and *IL12B* (r_s_ = 0.450, *p* = 0.011, Figure 2E) and *TGFB1* (r_s_ = 0.438, *p* = 0.014, Figure 2F).

### 3.3. Associations between Cytokine Gene Expression and RA Activity

**Figure 3:**
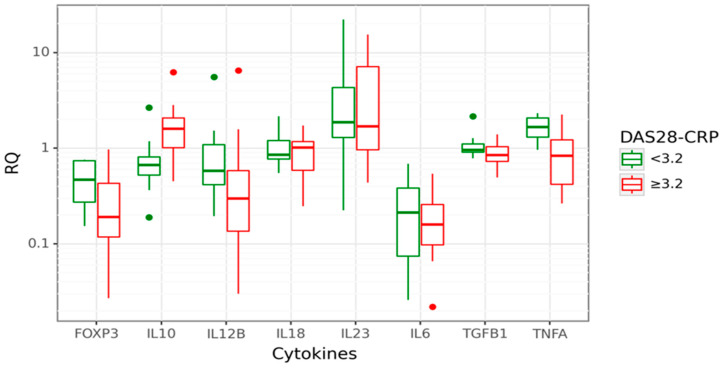
Box plot displaying gene expression in relative quantity (RQ) among RA patients with low (green) disease activity, DAS28-CRP < 3.2 (26% of all cases; n = 8) and high (red) disease activity, DAS28-CRP ≥ 3.2 (74% of all cases, n = 24). The line denotes the median values; boxes, the lower (Q1) and upper (Q3) quartiles; whiskers, minimum and maximum; and single point, outliers. The log scale is used for the Y-axis.

We analysed gene expression levels in relation to RA activity, measured by the Disease Activity Score 28, based on C reactive protein (DAS28-CRP). The RA patients with DAS28-CRP < 3.2 were classified as cases with low disease activity/remission, and RA patients with DAS28-CRP ≥ 3.2 were classified as cases with active disease. Data are shown in Figure 3.

**Figure 4:**
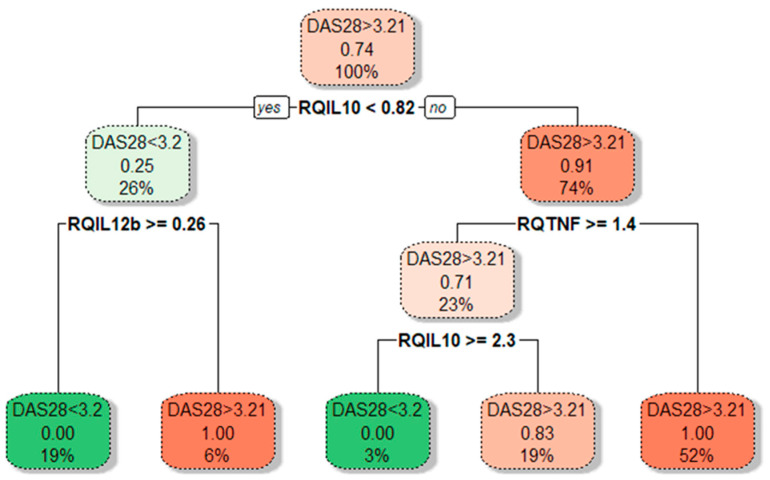
Tree plot showing decision boundaries distinguishing lower- and higher-activity RA patients (green and red) based on their cytokine gene expression levels. Tree plot uses thresholds of cytokine mRNA levels to produce optimal separation of RA patients in the final leaf nodes based on a boolean operator (YES/NO). The predominant class is depicted on every branch and leaf node; under it, the proportion of DAS28 > 3.2 from RA patients in that node; at the bottom, the percentage of patients in that particular branch or leaf node from the total patient count.

In cases with active disease (DAS28-CRP ≥ 3.2), approximately a 2-fold increase of *IL10* expression than in inactive RA (*p* = 0.006) was observed. Moreover, *IL10* gene expression was positively correlated with the DAS28-CRP score (r_s_ = 0.448; *p* = 0.012). A significantly decreased *FOXP3* gene expression (3.38-fold lower, *p* = 0.031) was observed in cases with active disease compared to inactive RA. This relation was even more profound among cases with the highest disease activity, DAS28-CRP ≥ 5.1 (8.85-fold lower *FOXP3* mRNA). However, this observation is limited due to the small number of patients with high disease activity, DAS28-CRP ≥ 5.1. Next, we performed a CART analysis to unravel that layer of interaction (Figure 4). 

**Figure 5:**
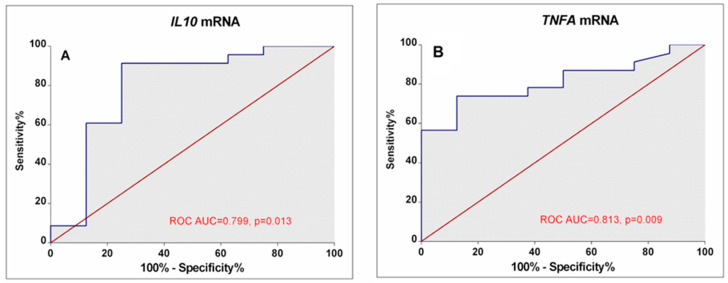
ROC Curves display the performance of IL10 (**A**) and TNFA (**B**) mRNA levels in discriminating between low disease activity/remission and high-activity RA patients. The area under the curve (ROC AUC) and *p*-value are depicted on the graphs.

Our results demonstrated that different disease activity cohorts could be separated based on different *IL10*, *TNFA*, and *IL12B* expression combinations. The first subgroup of lower-activity patients (19% of all RA patients) had lower than 0.82-fold *IL10* mRNA expression in combination with higher than 0.26-fold *IL12B* mRNA levels. Similarly, decision boundaries could also be derived for the higher-activity patients. The majority of higher-activity patients (52%) had simultaneously higher *IL10* expression and lower *TNFA* levels. In addition, ROC curve analysis (Figure 5) confirms the higher *IL10* (AUC: 0.799; 95%CI: 0.582–1.0; *p* = 0.013) and lower *TNFA* (AUC: 0.813; 95% CI: 0.662–0.963; *p* = 0.009) expression could be valuable discriminative factors for higher and lower RA activity.

### 3.4. Associations between Cytokine Gene Expression and RA Disease Characteristics

We analysed gene expression levels and other clinical characteristics of patients with RA, including their RF status (seropositive and seronegative), anti-CCP status (positive and negative), disease duration, disease onset, and medical treatment. We did not find significant associations between mRNA expressions of the analysed genes and RF status, anti-CCP status, disease duration, disease onset, and medical treatment (symptomatic or csDMARDs). Data are presented in Table 2.

### 3.5. Associations between Cytokine Serum Levels and RA Disease Characteristics

**Figure 6:**
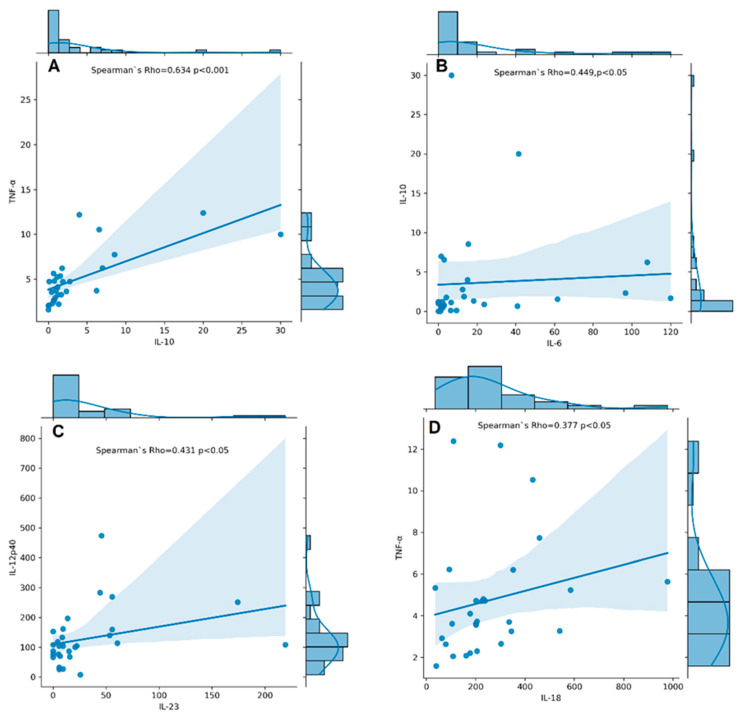
Correlation plots with histogram marginal densities and regression lines show correlations between serum cytokine levels IL-10 and TNF-α (**A**); IL-6 and IL-10 (**B**); IL-23 and IL-12p40 (**C**); IL-18 and TNF-α (**D**) in RA patients (n = 31). The histograms present data distribution. The Spearman’s rho–coefficient and *p*-values are presented.

Serum levels of IL-6, IL-10, IL-12p40, IL-17A, IL-18, IL-23, TNF-α, and TGF-β1 were measured in RA patients. TNF-α, IL-12p40, IL-17A, IL-18, IL-6, IL-23, IL-10, and TGF-β1 were detectable in all RA patients, whereas IL-6, IL-10, and IL-23 had detectability of 89%, 93%, and 83%, respectively. The concentrations of cytokines assayed in serum samples of RA patients are shown in Supplementary Table S1. We found a significantly strong correlation between serum levels of IL-10 and TNF-α (r_s_ = 0.634, *p* < 0.001, Figure 6A) and a moderate one between IL-10 and IL-6 (r_s_ = 0.449, *p* < 0.05, Figure 6B), IL-12p40 and IL-23 (r_s_ = 0.431, *p* < 0.05, Figure 6C), and IL-18 and TNF-α (r_s_ = 0.377, *p* < 0.05, Figure 6D).

There was no significant correlation between mRNA and serum level of any of the studied cytokines except a weak positive correlation between serum IL-12p40 and *IL12B* mRNA (r_s_ = 0.398, *p* = 0.033) among RA patients.

We performed a few descriptive analyses relating to the serum concentration of cytokines and RF status, the status of anti-CCP, disease duration, and DAS28-CRP. First, we start by analysing the serum concentration of cytokines and comparing their levels in patients with positive and negative RF statuses. We observed a significant elevation of serum IL-17 (mean ± SD, 23.09 ± 24.12 pg/mL vs. 3.23 ± 2.21 pg/mL, *p* = 0.004), IL-10 (mean ± SD, 5.32 ± 14.48 pg/mL vs. 0.69 ± 0.47 pg/mL, *p* = 0.017), and TNF-α (mean ± SD, 5.07 ± 3.12 vs. 3.31 ± 1.03, *p* = 0.031) in seropositive compared to seronegative patients.

We conducted a similar analysis to compare the relationship between cytokines and disease activity in two subgroups: low disease activity/remission, DAS28-CRP < 3.2 and active diseases, DAS28-CRP ≥ 3.2. Patients with active disease have higher serum levels of IL-6 compared to those with inactive disease (mean ± SD, 34.8 ± 59.8 vs. 3.34 ± 4.3, *p* = 0.004), as well as IL-18 (mean ± SD, 293.0 ± 218.5 vs. 166.0 ± 87.2, *p* = 0.068) and IL-23 (mean ± SD, 37.9 ± 56.9 vs. 11.9 ± 15.8, *p* = 0.062), although with borderline significance. In addition, a moderate correlation was found between the DAS28-CRP score and serum IL-6 levels (R = 0.463, *p* = 0.011) and TGF-β1 (R = 0.443, *p* = 0.016). Also, significantly elevated concentrations of IL-6 were found in patients with a disease duration of less than two years compared to those with longer disease duration (30.87 ± 40.59 pg/mL vs. 24.88 ± 56.02 pg/mL, *p* = 0.031).

We did not find a significant difference among RA patients in our analysis of the relationship between serum concentration of cytokines, anti-CCP status (positive or negative), and medical treatment (symptomatic or csDMARDs).

## 4. Discussion

Our previous studies demonstrated elevated serum inflammatory cytokines in women with RA with altered cytokine production depending on different treatment regimens [13]. However, investigation of immunoregulatory IL-10 and TGF-β1 in the same cohort revealed down-regulated systemic TGF-β1 but elevated IL-10 [8]. Also, we found that some single-nucleotide polymorphisms on promoter regions on *IL10* and *IL12B* genes regulate serum cytokine expression and influence disease occurrence [8,9]. In this regard, the present study exploring the cytokine mRNA expression in the group of RA patients is a continuation of our previous investigations to understand better the involvement of inflammatory and regulatory cytokines in disease development.

Our current study provides evidence for an imbalance in pro-inflammatory and immunoregulatory gene expression in peripheral blood cells of patients with RA that may contribute to the perpetuation of inflammation and tissue damage observed in RA. Higher expression of *IL23A* mRNA and lower *IL12B* expression confirms the crucial role of the IL-23/Th17 axis compared to IL-12/Th1. In response to IL-23- and IL-18-mediated activation, effector Th17 cells produce IL-17, IL-6, and TNF-α, thus contributing to chronic inflammation and tissue damage characteristics of the disease [26,27]. In addition, we observed a significant decrease of *IL6* and *FOXP3* mRNAs in RA patients’ blood compared to healthy controls, which could be related to the reduced regulatory capacity of forkhead box P3 protein Foxp3-expressing Treg cells in RA. The conducted correlation analyses also revealed a positive correlation between *FOXP3* and *TGFB1* expression and an unexpectedly strong positive correlation between the expression of *FOXP3* and *TNFA*. A positive correlation between the gene expression of *TNFA* and *FOXP3* in the blood cells of RA patients might seem surprising due to the antagonistic roles typically attributed to both genes in RA. However, several potential reasons could underlie this correlation. Both Tregs and Th17 sub-populations are influenced by TNF-α signalling through the engagement of TNFR1 and TNFR2. TNFR1 is involved in the development of effector T cells and is expressed by both Tregs and Th17 subpopulations, whereas TNFR2 is the most important receptor for TNF-α-dependent regulation and activation of Tregs, which emphasises the dual role of TNF-α in the Th17/Treg axis [18,28]. Altered immune regulation in RA may lead to pro-inflammatory and anti-inflammatory responses. The decreased expression of both *TNFA* and *FOXP3* might reflect the dysregulation as a result of attempts to control chronic inflammation in RA. Another possibility is a breaking of post-transcriptional regulation in the synthesis of these proteins, for example, through abnormal synthesis of microRNAs involved in the regulation of translation [29]. The role of miRNA in the pathogenesis of RA concerning new treatment approaches has recently been extensively investigated [30].

In the context of RA, the levels of TNF-α and Foxp3 can influence various aspects of the immune response, including autoantibody production, cell death (apoptosis), and the formation of neutrophil extracellular traps (NETs) through a process called NETosis. TNF-α contributes to activating B cells, which are responsible for producing antibodies. In RA, including participants in our study, autoantibodies like RF and anti-CCP are commonly elevated. Elevated TNF-α levels might stimulate B cells, leading to increased production of these autoantibodies [31]. TNF-α is involved in inducing apoptosis in certain cell types, including some immune cells. However, in RA, a complex balance exists, as TNF-α can also promote the survival of inflammatory cells, contributing to chronic inflammation and tissue damage. TNF-α can stimulate neutrophils to undergo NETosis, exacerbating the inflammatory response [32]. Foxp3, on the other hand, is important for maintaining tolerance to self-antigens potentially impacting autoantibody production and mitigating excessive cell death or NETosis. Our data regarding the correlation of TNF-α, IL-17, and IL-10 and RF antibody production complies with the abovementioned mechanisms.

The mRNA expression profile of the study group might be influenced by the diversity of RA patients in terms of disease severity, duration, treatment history, or other factors. Respectively, our further subgroup analyses, according to disease activity, showed that the RA activity was associated with higher *IL10* expression and lower *TNFA*. In cases with active disease with DAS28-CRP ≥ 3.2, approximately a 2-fold increase of *IL10* expression was observed. Based on the performed CART analysis, different disease activity cohorts could be separated based on different *IL10*, *TNFA*, and *IL12B* expression combinations. The majority of higher-activity patients (52%) had higher *IL10* expression combined with lower *TNFA* levels. CART results described herein complied with our prior notion. The non-linear relationships between cytokine pathways are also integral to RA pathogenesis. Therefore, CART analysis is quite a helpful technique to localise hidden subgroups and correlations that display complex cytokine biology. In addition, ROC analysis showed that higher *IL10* and lower *TNFA* mRNA could be used as valuable biomarkers for RA disease activity.

Previous studies on peripheral blood of RA patients have reported increased IL-10 production and a higher proportion of CD4^+^IL10^+^ and CD4^+^CD25^int^IL10^+^ cells in patients with moderately active disease. The authors suggested that during active disease, these cell subsets produce progressively larger amounts of IL-10 to regulate inflammation in response to disease activation [33]. In cases of active RA, elevated *IL10* expression might represent a compensatory response to counteract excessive inflammatory signalling, which includes down-regulating the expression of *TNFA* to reduce inflammation and tissue damage. Thus, therapeutic manipulation of IL-10 in RA, including administration of recombinant IL-10 or IL-10 fusion antibody in combination with MTX, has been applied in preclinical and clinical studies [34,35]. A promising approach for targeting inflammation in RA in preclinical trials was the administration of IL4-10 fusion protein as a disease-modifying osteoarthritis drug due to its known synergic chondroprotective and anti-inflammatory properties [16]. Another study has shown that peripheral blood monocyte cells (PBMCs) derived from active RA patients contained characteristic hypomethylation of CpG sites at −145 bp within the proximal promoter of the *IL10* gene and was associated with elevated IL-10 expression [36]. A recent study gives experimental evidence that enhanced IL-10 production associated with down-regulation of mir-96 can promote TNF-α- mediated apoptosis of human fibroblast-like synoviocytes [37].

In addition, some reports showed that methotrexate therapy altered DNA methylation status that could be distinct in T lymphocytes obtained by synovium or peripheral blood of RA patients [38], as well as could be cell-specific [39]. Also, methotrexate as a monotherapy reduces the mRNA expression of *IL6* and, combined with corticosteroids, decreases expression levels of *IL18* in PBMCs of RA patients [40]. Most of the participants in our study had used these treatments during their enrollment, and the therapeutic regimen could influence the gene expression profile.

Another result of our study concerns differences between serum level and mRNA synthesis of investigated cytokines in RA patients. Observed downregulation of *IL*6 and lack of upregulation of *TNFA* in PBMC can be due to either “direct” or “indirect” mechanisms, including post-transcriptionally regulated expression of a protein that, in turn, influences the level of the cytokine [41]. Indirect regulation by miRNAs transported with extracellular vesicles in blood can alter cytokine production in peripheral immune cells. In addition, the excessive or persistent cytokine production in RA is mainly performed by locally activated immune cells in synovia.

The increasingly accumulating evidence for the complexity of gene expression regulation, which may result in poor mRNA–protein correlations, dictated the new direction in our investigations of RA, studying the gene expression at the mRNA level in peripheral blood. Detecting changes in narrow panel key cytokines at mRNAs in patient blood by qPCR might provide a sensitive, low-cost, and simple diagnostics, screening or monitoring approach. In addition, the study provides insights into the differential expression of key cytokines and affected molecular mechanisms in the immunopathogenesis of RA patients.

### Limitations

However, our study has some limitations that could influence the interpretability of our results. First to be mentioned is the relatively small sample size. Second, the heterogeneity of the study sample regarding clinical presentation and therapeutic regimen (typical for RA) combined with the lack of longitudinal follow-up obscures the complex cytokine-induced intracellular network even more. Therefore, future studies on a large number of samples and longitudinal data are warranted to confirm the obtained data.

## 5. Conclusions

In summary, dysregulated gene expression of studied cytokines in RA patients’ peripheral blood cells reflects the imbalance in the Th1/Th17/Treg axis in this chronic progressive autoimmune disorder. The main characteristic includes a predominant overexpression of *IL23A* and downregulation of *IL12B*, *IL6*, and *FOXP3*. Also, different disease activity cohorts could be separated based on distinguishing *IL10*, *TNF*A, and *IL12B* expression combinations. Active RA was associated with an elevated *IL10* combined with lower *TNFA* and *IL12B* expression.

## Figures and Tables

**Table 1:** Demographic characteristics and clinical data of RA patients and study controls.

	RA	Controls	*p*-Value
n	31	21	
Sex, n (%)			1.0
Male	5 (16.1)	3 (14.3)	
Female	26 (83.9)	18 (85.7)	
Age (mean ± SD; years)	43.1 ± 14.2	39.3 ± 12.2	0.324
Disease duration (mean ± SD; years)	9.6 ± 8.5		
RF-positive patients; n (%) (RF positivity > 14.0 U/mL)	25 (81)		
Anti-CCP-positive patients; n (%) (anti-CCP positivity > 5 RU/mL)	22 (71)		
CRP (mean ± SD; mg/L) (CRP reference range 0–6 mg/L)	19.2 ± 39.64		
DAS28-CRP (mean ± SD)	4.1 ± 1.05		
<3.2; n (%)	8 (26)		
≥3.2; n (%)	23 (74)		
Therapy, n (%)			
Symptomatic	9 (29)		
csDMARDs	22 (71)		

CCP, cyclic citrullinated peptide autoantibodies; CRP, C reactive protein; DAS28-CRP, Disease Activity Score 28 calculated using C reactive protein; RA, rheumatoid arthritis; RF, rheumatoid factor; SD, standard deviation; csDMARDs conventional synthetic disease-modifying anti-rheumatic drugs.

**Table 2:** mRNA expression in relation to RA disease characteristics.

mRNA (RQ)	*IL6*	*IL10*	*IL12B*	*IL18*	*IL23*	*TNFA*	*TGFB1*	*FOXP3*
Disease onset	0.25 ± 0.24	1.33 ± 0.71	0.92 ± 1.56	0.91 ± 0.37	4.74 ± 6.0	1.28 ± 0.67	0.90 ± 0.23	0.40 ± 0.28
<40 years (n = 16)	0.21 ± 0.17	1.71 ± 1.45	0.82 ± 1.39	1.05 ± 0.50	4.62 ± 5.5	0.98 ± 0.65	0.98 ± 0.41	0.28 ± 0.23
>40 years (n = 15)	*p* = 0.833	*p* = 0.833	*p* = 0.698	*p* = 0.495	*p* = 0.711	*p* = 0.299	*p* = 0.892	*p* = 0.247
Disease duration	0.28 ± 0.21	1.01 ± 0.26	0.39 ± 0.50	0.76 ± 0.38	4.74 ± 6.0	0.74 ± 0.43	0.81 ± 0.19	0.27 ± 0.19
<2 years (n = 7)	0.22 ± 0.22	1.66 ± 1.24	1.00 ± 1.62	1.04 ± 0.45	4.62 ± 5.5	1.25 ± 0.68	0.98 ± 0.35	0.37 ± 0.27
>2 years (n = 24)	*p* = 0.692	*p* = 0.167	*p* = 0.182	*p* = 0.495	*p* = 0.139	*p* = 0.085	*p* = 0.216	*p* = 0.473
RF	0.14 ± 0.10	1.25 ± 0.95	1.54 ± 2.44	0.91 ± 0.37	2.79 ± 2.52	1.53 ± 0.80	1.15 ± 0.55	0.43 ± 0.25
negative (n = 6)	0.25 ± 0.22	1.58 ± 1.17	0.70 ± 1.13	1.05 ± 0.50	5.14 ± 6.18	1.04 ± 0.61	0.89 ± 0.23	0.32 ± 0.26
positive (n = 25)	*p* = 0.513	*p* = 0.417	*p* = 0.698	*p* = 0.295	*p* = 0.764	*p* = 0.153	*p* = 0.563	*p* = 0.104
Anti-CCP	0.28 ± 0.28	1.95 ± 1.71	1.13 ± 2.06	1.16 ± 0.57	4.45 ± 5.78	1.30 ± 0.81	1.01 ± 0.49	0.39 ± 0.27
negative (n = 9)	0.22 ± 0.19	1.34 ± 0.77	0.76 ± 1.17	0.90 ± 0.37	4.78 ± 5.79	1.02 ± 0.58	0.91 ± 0.23	0.33 ± 0.26
positive (n = 22)	*p* = 0.604	*p* = 0.428	*p* = 0.881	*p* = 0.273	*p* = 0.881	*p* = 0.292	*p* = 0.949	*p* = 0.507
Therapy	0.23 ± 0.18	2.08 ± 1.65	1.35 ± 2.0	0.92 ± 0.43	6.76 ± 6.02	1.22 ± 0.75	0.99 ± 0.25	0.42 ± 0.30
Symptomatic (n = 9)	0.24 ± 0.26	1.3 ± 0.78	0.69 ± 1.19	1.03 ± 0.46	3.77 ± 5.6	1.1 ± 0.66	0.92 ± 0.36	0.29 ± 0.22
csDMARDs (n = 22)	*p* = 0.903	*p* = 0.334	*p* = 0.294	*p* = 0.468	*p* = 0.360	*p* = 0.884	*p* = 0.381	*p* = 0.152

Data are presented as mean ± SD; CCP, cyclic citrullinated peptide autoantibodies; csDMARDs conventional synthetic disease-modifying anti-rheumatic drugs; RA, rheumatoid arthritis; RF, rheumatoid factor; SD, standard deviation.

## Data Availability

The data presented in this study are available upon request from the corresponding author. The data are not publicly available due to local regulations and hospital restrictions.

## Supplementary Materials

The following supporting information can be downloaded at: https://www.mdpi.com/article/10.3390/cimb46030167/s1, Table S1: Serum levels of study cytokines in RA patients.
