# Supplementary material for: An Elevated IL10 mRNA Combined with Lower TNFA mRNA Level in Active Rheumatoid Arthritis Peripheral Blood
Source: Curr Issues Mol Biol. 2024 Mar 20;46(3):2644–57. doi: 10.3390/cimb46030167 (PMC10969346; doi:10.3390/cimb46030167)
Supplement: Supplementary file 1 [file cimb-46-00167-s001.zip › cimb-2910868-supplementary.pdf]

Supplementary Table S1. Serum levels of study cytokines in RA patients

Cytokine levels	Mean ± SD	Q1 (p25)	Q2 (Median)	Q3 (p75)	Range
IL-6 pg/ml	26.12 ± 52.5	1.81	6.57	20.96	0.00 - 258.99
IL-10 pg/ml	8.23 ± 25.02	0.66	1.22	3.38	0.00 - 125.55
IL-12p40 pg/ml	131.64 ± 99.92	70.06	106.76	160.00	7.88 - 473.65
IL-17A pg/ml	35.54 ± 72.71	3.81	11.69	25.15	1.31 - 361.31
IL-18 pg/ml	260.0 ± 201.64	108.86	205.64	342.07	35.64 - 977.00
IL-23 pg/ml	30.74 ± 50.13	5.43	9.36	45.02	0.00 - 219.00
TNF-α pg/ml	4.76 ± 2.81	2.79	3.74	5.5	1.59 - 12.38
TGF-β ng/ml	15.13 ± 9.9	7.68	13.79	21.5	2.30 - 45.59
